# CAMSAP2 promotes colorectal cancer cell migration and invasion through activation of JNK/c-Jun/MMP-1 signaling pathway

**DOI:** 10.1038/s41598-022-21345-7

**Published:** 2022-10-07

**Authors:** Xiaojuan Wang, Yumin Liu, Yawen Ding, Gang Feng

**Affiliations:** 1 Department of Oncology, Wuhan Fourth Hospital, Puai Hospital, Tongji Medical College, Huazhong University of Science and Technology, 473 Hanzheng Street, Wuhan, 430000 Hubei China; 2 Department of Obstetrics and Gynecology, Wuhan Hankou Hospital, Wuhan, 430010 Hubei China

**Keywords:** Cancer, Oncology

## Abstract

CAMSAP2 has been reported to act as an oncogene in hepatocellular carcinoma. However, the expression CAMSAP2 and its potential roles in colorectal cancer remain unclear. In this study, qRT-PCR and immunoblotting analysis were used to detect the mRNA and protein levels of CAMSAP2 in colorectal cancer tissues and cell lines. Wound-healing, transwell migration and invasion assay were performed to determine whether CAMSAP2 promotes the capabilities of migration and invasion of colorectal cancer cells. The results showed that CAMSAP2 was highly elevated in colorectal cancer tissues and cell lines. Moreover, the high CAMSAP2 expression was positively correlated with tumor invasion depth, lymph node metastasis, distant metastasis, and the poor prognosis of colorectal cancer. Additionally, ectopic expression of CAMSAP2 in colorectal cancer cells promoted the migration and invasion in vitro and enhanced the lung metastasis in nude mice. Conversely, silencing CAMSAP2 resulted in an opposite phenomenon. By gain- and loss-of function experiments, we demonstrated that MMP-1 was a substantial downstream target of CAMSAP2, and it played a crucial role in regulating the migration and invasion induced by CAMSAP2 in colorectal cancer cells. Mechanistically, CAMSAP2 promoted the activation of JNK/c-Jun signaling pathway and subsequently upregulated the transcription activity of MMP-1. Taken together, our findings demonstrated that CAMSAP2 promoted colorectal cancer cell migration, invasion and metastasis through activation of JNK/c-Jun/MMP-1 signaling pathway, indicating CAMSAP2 is a promising therapeutic target for the treatment of metastatic colorectal cancer patients.

## Introduction

As a common malignant tumor in digestive system, colorectal cancer remains a substantial public health challenge all over the world in the past 30 years^1^. Colorectal cancer as the second leading cause of cancer-related mortality in 2020, with an estimated 935,000 deaths, represents 9.4% of all cancer cases worldwide^2^. In addition, it is the third cause of global cancer incidence, with more than 1.9 million new colorectal cancer (including anus) cases, representing 10.0% of all cancer cases^2^. Currently, although there are numerous therapeutic treatments including surgery, cytotoxic chemotherapy, immunotherapy, targeted therapy, radiation, and combination strategies have been used for the patients with colorectal cancer, the prognosis of colorectal cancer patients remains unsatisfactory^3, 4^. Moreover, 20% of patients with colorectal cancer present with metastasis at the time of initial diagnosis, and of those patients with primary disease approximately 50% will eventually develop metastatic disease^4^. More seriously, the overall 5-year survival rate for those patients with metastatic colorectal cancer is only 14%^4, 5^. Therefore, it is critical to further elucidate the molecular mechanisms underlying colorectal cancer metastasis to improve the life of patients with colorectal cancer.

CAMSAPs (calmodulin-regulated spectrin-associated proteins), including three homologues (CAMSAP1, CAMSAP2 and CAMSAP3), has been demonstrated to specifically recognize microtubule minus ends and control their dynamics in different animal systems^6, 7^. As one important membrane of this family, the major function of CAMSAP2 is to generate strongly stabilized microtubule lattices to serves as “seeds” for noncentrosomal microtubule outgrowth^8–10^. In addition, CAMSAP2 has a strong effect on microtubule dynamics to specifically inhibit microtubule minus-end polymerization and catastrophes^10^. Vice versa, the depletion of CAMSAP2 strongly reduced the number of non-centrosomal microtubules. In addition, silencing CAMSAP2 also drastically reduced the microtubule density, increased microtubule growth rate and longer EB1 comets, and inhibited cell migration in epithelial cells. Li et al. first reported that CAMSAP2 was dramatically upregulated in hepatocellular carcinoma (HCC) compared with adjacent nontumor tissues. Additionally, CAMSAP2 high-expression correlated with multiple tumors, increased tumor size, microvascular invasion, poor tumor differentiation, and a higher tumor-nodule-metastasis stage and predicted poor prognosis in HCC^11^, indicating that CAMSAP2 may play a crucial role in promoting HCC progression and metastasis. Indeed, ectopic expression of CAMSAP2 markedly enhanced HCC cell migration, invasion and metastasis in vitro and in vivo, whereas CAMSAP2 depletion had the opposite effect^11^. Recently, Lv. et al. also discovered that CAMSAP2 was highly elevated in human non-small-cell lung cancer (NSCLC) tumors tissue and cell lines, compared to the corresponding normal tissues and cells^12^. Moreover, elevated expression of CAMSAP2 completely reversed the migration and invasion inhibited by miR-2355-5p NSCLC cells^12^. However, the expression of CAMSAP2 and its functions in colorectal cancer cell migration and invasion remains unclear.

In this study, we detected the expression and investigated the potential role and molecular mechanism of CAMSAP2 in colorectal cancer. The results revealed that CAMSAP2 is highly expressed in colorectal cancer cells and clinical samples. Further investigations demonstrated that CAMSAP2 promotes the expression of MMP-1 via activation of JNK/c-Jun signaling pathway, resulting in the migration, invasion and metastasis of colorectal cancer cells, suggesting CAMSAP2 is a promising therapeutic target for the treatment of metastatic colorectal cancer patients.

## Methods and materials

### Cell lines and cell culture

The human colorectal cancer cell lines (SW-480, HCT-8 and SW-620) obtained from the Shanghai Cell Bank, Chinese Academy of Sciences (Shanghai, China). The human normal colorectal epithelial cells (FHC) were purchased from American Type Culture Collection (ATCC). HCT-8 cells were cultured in RPMI-1640 with 10% fetal bovine serum including 1% antibiotics. Dulbecco’s Modified Eagle’s Medium (DMEM, Gibco) supplemented with 1% antibiotics and 10% fetal bovine serum (FBS) were used to culture the FHC, SW-480 and SW-620 cells, the culture incubator was sterile at 37 ℃ with a humidified atmosphere of 5% CO_2_. All cell lines were confirmed by short tandem repeat profiling and tested for mycoplasma using Mycoplasma Detection Kit (Thermo Fisher Scientific, San Jose, CA, USA).

### Tissue samples and clinical data collection

The 76 samples of human colorectal cancer and their adjacent non-tumor tissues were obtained from patients during operation at Wuhan Fourth Hospital of Huazhong University of Science and Technology (Nanjing, China). All collected tissue samples were immediately snap frozen in liquid nitrogen and stored at −80 °C until required. The patient characteristics were listed in Supplementary Table S1. This study was approved by the ethics committee on Human Research of the Wuhan Fourth Hospital of Huazhong University of Science and Technology. Moreover, we confirm that all methods were performed in accordance with the relevant guidelines and regulations. Written informed consent was obtained from all patients.

### Reagents and DNA constructs

SP600125 were purchased from Selleck (Shanghai, China), dissolved in dimethyl sulfoxide (DMSO) for stock solution at 20 mM and stored at −20 ℃. psPAX2 (packaging plasmid, #12260) and pMD2.G (envelope plasmid, #12259) were obtained from Addgene (Cambridge, MA). Human CAMSAP2, MMP-1 or c-Jun was cloned into the pCMV-Flag-His-PuroR vector by Transheep (Shanghai, China) by using ClonExpress MultiS One Step Cloning kit (Vazyme, Nanjing, China). Human CAMSAP2 or MMP-1 specific shRNAs were obtained from Sigma-Aldrich (Shanghai, China). The pG3-Luc which contains MMP-1 gene promoter upstream of the firefly luciferase gene and and pRL-TK renilla luciferase reporter constructs were from Promega (Shanghai, China).

### RNA extraction and quantitative real-time polymerase chain reaction (qRT-PCR) assay

Total RNA was extracted using TRIzol (Invitrogen, Carlsbad, CA), and reverse transcription reactions were performed using Superscript III reverse transcriptase (Invitrogen, Carlsbad, CA) and random primers according to the manufacturer’s instructions^13^. Target genes were measured using SYBR Green PCR Master Mix (Applied Biosystems) on an ABI Prism 7900 sequence detection system (Applied Biosystems). The conditions of PCR were: 40 cycles of 95 °C for 20 s, 60 °C for 30 s, and 72 °C for 30 s, and one cycle of 72 °C for 10 min. GAPDH was used as an internal control for normalization and the relative expression of mRNA abundance was calculated using the 2^−ΔΔCT^ method. The sequences of primers used in this study are listed in Supplementary Table S2.

### Western blot analysis

Western blot analysis was conducted as previous described^3^. Briefly, after washing three times with ice-cold phosphate buffer saline (PBS), cells were harvested and disrupted in RIPA lysis buffer (Beyotime, Shanghai, China). Cell debris was removed via centrifugation at 12, 000 rpm/min at 4 °C for 20 min. The total protein concentration was quantified using Pierce BCA Protein Assay Kit (23225, Pierce, Washington, USA). For Western blot analysis, protein was separated using SDS-PAGE and transferred onto PVDF membranes (Millipore, Billerica, MA, USA). The PVDF membranes were blocked in tris–phosphate-buffered saline (TPBS) supplemented with 5% fat-free milk at room temperature for 2 h and then incubated with the indicated specific primary antibodies at 4 °C for overnight. After washing three times with TPBS, the membrane was treated with the corresponding horseradish peroxidase (HRP)-conjugated secondary antibodies for 1 h at room temperature. Protein bands were visualized using enhanced chemiluminescence (Amersham; Buckinghamshire, UK). The antibodies used in this study were listed as follows: CAMSAP2 (1:1000, Proteintech, 17880-1-AP), MMP-1 (E9S9N) (1:1000, Cell Signaling Technology, 54376), p-JNK (T183/Y185) (G9) (1:1000, Cell Signaling Technology, 9255), JNK (1:1000, Cell Signaling Technology, 9252), p–c-Jun (Ser73) (D47G9) (1:1000, Cell Signaling Technology, 3270), c-Jun (60A8) (1:1000, Cell Signaling Technology, 9165), Actin (1:1000, Proteintech, 23660-1-AP), HRP-conjugated Affinipure Goat Anti-Mouse IgG (H + L) (1:4000, Proteintech, SA00001-1) and HRP-conjugated Affinipure Goat Anti-Rabbit IgG (H + L) (1:4000, Proteintech, SA00001-2).

### Plasmid transfection

For gene overexpression experiments, colorectal cancer cells were transfected with the indicated gene expression plasmid or empty vector using Lipofectamine 3000 (Invitrogen, Carlsbad, CA) according to the manufacturer’s instructions^14^. The lentivirus were produced by transfection in 293 T cells with control shRNA (shNC) or specific shRNAs targeting CAMSAP2 or MMP-1 together pMD2.G and psPAX2 using Lipofectamine 3000. Viruses were collected after 48 h transfection and purified using 0.45-μm filters. After infection with the indicated lentivirus in the presence of 8 μg/mL polybrene, SW-480 and SW-620 cells were selected with 1.5 μg/mL puromycin for 2 weeks. QRT-PCR and Western blot analysis were used to detect the overexpression or knockdown efficiency.

### Transwell migration and invasion assays

Transwell migration and invasion assays were performed using transwell with polyethylene terephthalate membranes (24-well inserts, 8.0 μm, Corning)^15^. For migration assay, 200 μL cell suspensions contained 5 × 10^4^ cells was loaded into the upper chamber of a transwell. Next, 600 μL DMEM medium with 10% FBS was placed into the bottom of the well as a source of chemo-attractants. 24 h later, the cells on the lower surface of the insert were fixed with methanol and staining with 0.5% crystal violet. For transwell invasion assay, a similar procedure was performed as the above description except that 1 × 10^5^ cells was loaded into upper chamber pre-coated with matrigel (BD Biosciences, CA, USA). Staining cells were visualized and photographed using a CKX41 microscope (Olympus, Japan). Randomly selected five fields and counted the cells, experiments were repeated three times and the data are presented as the means ± SD.

### Luciferase assays and plasmid construction

The pGL3-basic firefly luciferase reporter (GeneCreat, China) containing the MMP-1 gene promoter and pRL-TK renilla luciferase control reporter vector were used. 50 ng reporter plasimd and 20 ng renilla luciferase were co-transfected into the indicated tumor cells for 24 h by using Lipofectamine 3000 (Invitrogen) according to the manufacturer’s instructions^13^. The experiments were performed in triplicate. Then, cells were lysed by passive lysis buffer (Promega, Madison, WI) and reporter activities were investigated by the Dual-Luciferase Reporter Assay System (Promega). Renilla luciferase activity was normalized to firefly luciferase activity.

### Wound healing assay

Colorectal cancer cells (SW-620 and SW-480) were seeded in six-well plates (Corning, NY) and grown to confluence. The cells were washed with PBS and replaced with serum-free DMEM medium after scratched with a 200 μL sterile micropipette tip. The wounded area was recorded using an inverted phase-contrast microscope. The wound-healing rate was calculated as: ([wound width at 0 h − width at 24 h]/width at 0 h) × 100%^16^.

### ChIP

The ChIP assay was performed using the EZ-ChIP Kit (EMD Millipore) according to the manufacturer’s instructions. Briefly, cells were crosslinked in 1% formaldehyde for 10 min at room temperature and then quenched with glycine. The crosslinked chromatin was fragmented to 200–800 bp by sonication. The supernatant was subjected to immunoprecipitated using corresponding antibodies as indicated (anti-c-Jun and rabbit IgG) and Protein A/G agarose (60 μl of a 50% slurry) at 4 °C overnight. The primers for amplification of purified DNA are listed in Supplementary Table S3. The PCR conditions were 5 min at 94 °C for initial denaturation, 30 s at 94 °C for denaturation, 30 s at 60 °C for annealing, 30 s at 72 °C for extension, repeat for total 35 cycles, 72 °C for 5 min for final extension as previously described^17^. Fold enrichment was calculated using the ΔΔCt method.

### Mouse model of tumor metastasis

Our protocol used for animal was approved by the Institution Animal Care and Use Committee of Huazhong University of Science and Technology, and all animal experiments were carried out according to the Guide for the Care and Use of Laboratory Animals, and in strict accordance with the People’s Republic of China Legislation Regarding the Use and Care of Laboratory Animals. We also confirmed that the mouse experiments were carried out in compliance with the ARRIVE guidelines^18^. Male athymic BALB/c nude mice (18–20 g, 5 weeks old) were purchased from Beijing Vital River Laboratory Animal Technology Co., Ltd. All mice were housed in a controlled environment at 23 ± 2 °C, 40–60% humidity under a 12 h dark/light cycle with free access to irradiated food and sterile water.

SW620 cells (2 × 10^6^) were suspended in 200 μL PBS and then slowly injected into the tail vein of the mice (n = 6 per group) as previously described^19^. Six weeks later, the animals were anesthetized with isoflurane before killed by cervical dislocation. The lungs were dissected and fixed in Bouin’s solution (formaldehyde:acetic acid:saturated picric acid = 5:1:15) overnight. The metastatic foci on the surface of the lung in each mouse were counted, and the presence of tumor lesions within the lungs was confirmed by haematoxylin and eosin (H&E) staining.

### Statistical analysis

All statistical analyses were performed using GraphPad Prism software version 7.00 for Windows (GraphPad Prism Software, San Diego, CA, USA) and reported as the means ± standard deviation (SD). Student’s t-test was used to evaluate the difference between two different groups. In addition, Statistical significance between more than two groups was estimated by one-way analysis of variance (ANOVA). A value of *P* < 0.05 was considered significant.

## Results

### CAMSAP2 was elevated in colorectal cancer

**Figure 1:**
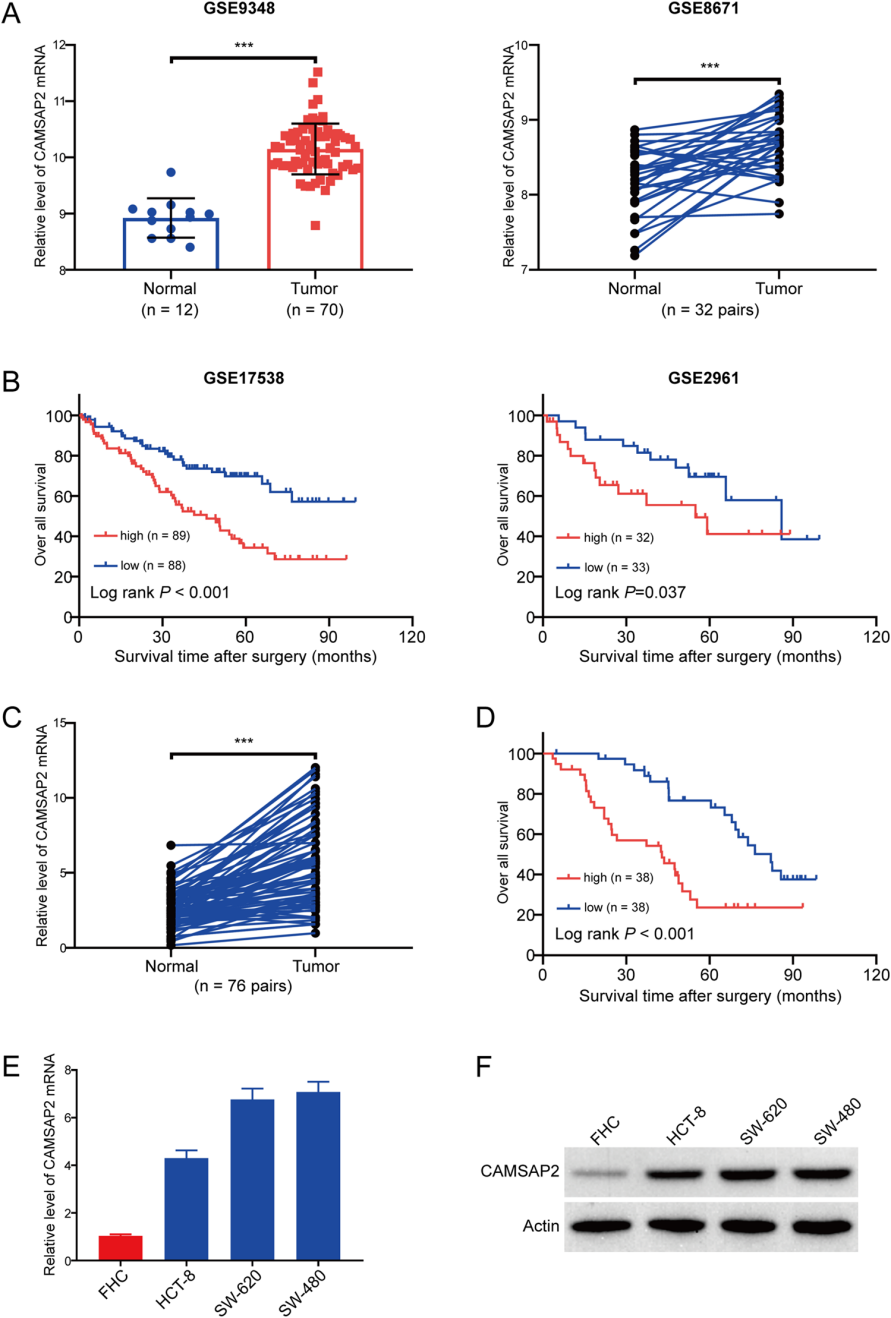
CAMSAP2 was highly expressed in colorectal cancer and is associated with poor prognosis. (**A**) The mRNA level of CAMSAP2 in GSE9348 (left panel) and GSE8671 (right panel) cohorts. (**B**) Influence of CAMSAP2 expression on overall survival by Kaplan–Meier analysis in GSE17538 (n = 177) and GSE2961 (n = 65) cohorts. (**C**) qRT-PCR analysis of CAMSAP2 mRNA in 76 pairs of colorectal cancer and the adjacent normal tissues. (**D**) Kaplan–Meier survival analysis of colorectal cancer patients’ overall survival based on the mRNA level of CAMSAP2 in our cohort (n = 76). (**E**) The mRNA expression of CAMSAP2 in colorectal cancer cells (HCT-8, SW-620 and SW-480) and FHC cells was detected by using qRT-PCR analysis. (F) Western blot analysis of CAMSAP2 protein in FHC and colorectal cancer cell lines. ***P < 0.001.

To investigate the expression of CAMSAP2 in colorectal cancer, we first analyzed two independent microarray datasets downloaded from GEO. As illustrated in Fig. 1A, the expression of CAMSAP2 mRNA was elevated in colorectal cancer, compared to the normal para-cancerous tissues. In addition, the result of Kaplan–Meier survival analysis indicated that patients with high CAMSAP2 expression had a poor prognosis in colorectal cancer patients derived from two independent microarray datasets downloaded from GEO (Fig. 1B). We then detected the expression of CAMSAP2 in 76 paired colorectal cancer tissues and adjacent tissues by using qRT-PCR. As shown in Fig. 1C, the level of CAMSAP2 in colorectal cancer tissues was much higher than that in the corresponding normal tissues. In addition, Kaplan–Meier survival analysis also revealed that a higher expression of CAMSAP2 corrected with reduced overall survival in colorectal cancer patients (*P* < 0.001) (Fig. 1D). Next, we performed chi-square test to analyze the correlation between CAMSAP2 expression and clinic-pathological features. As illustrated in Supplementary Table S1, the expression of CAMSAP2 was positively associated with tumor invasion depth (P = 0.0099), lymph node metastasis (P = 0.0359), distant metastasis (P = 0.0042) and TNM stage (P = 0.0249). Subsequently, we examined CAMSAP2 expression in colorectal cancer cell lines using qRT-PCR. The results showed that the mRNA level of CAMSAP2 was much higher in colorectal cancer cells then that in the human normal colorectal FHC cells (Fig. 1E). Consistently, Western blot analysis also revealed that CAMSAP2 was highly expressed in all three tested colorectal cancer cell lines, compared to the FHC cells (Fig. 1F).

### CAMSAP2 promoted colorectal cancer metastasis in nude mice

**Figure 2:**
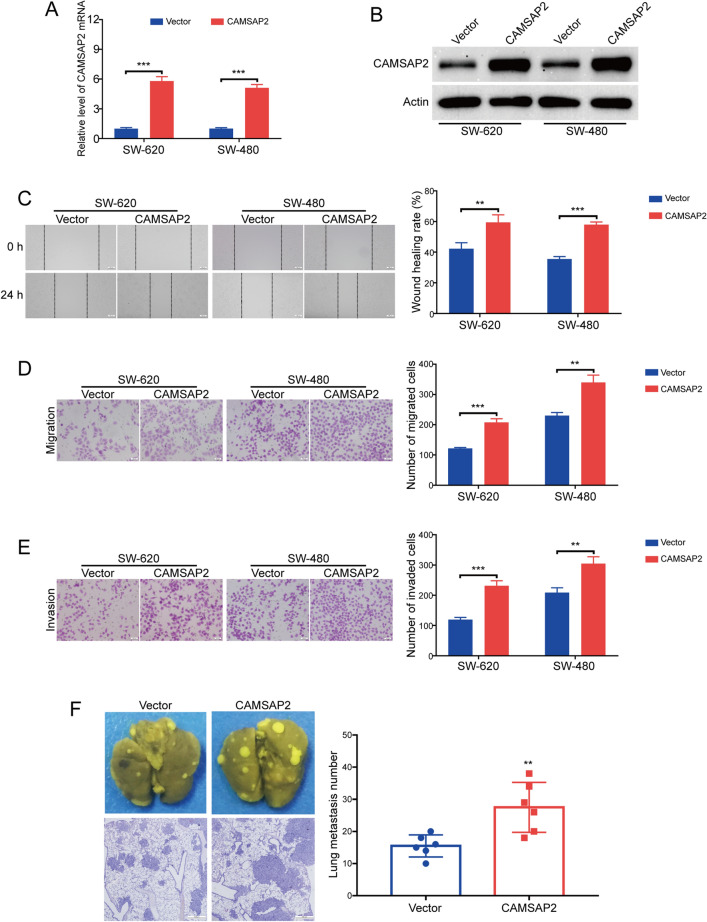
Overexpression of CAMSAP2 promoted migration and invasion in colorectal cancer cells. (**A**) qRT-PCR analysis of CAMSAP2 mRNA in SW-620 and SW-480 cells transduced with CAMSAP2 plasmid (CAMSAP2) or control plasmid (Vector). (**B**) Immunoblotting analysis validated that CAMSAP2 was overexpressed in SW-620 and SW-480 cells transduced with CAMSAP2 plasmid. (**C**) Wound healing assay showed overexpression of CAMSAP2 enhanced cell migration in colorectal cancer cells. Left: representative images of wound scratch. Right: histograms represent the analysis of the wound healing rate. Scale bar: 100 μm. (**D**) Overexpression of CAMSAP2 promoted the migration of colorectal cancer cells examined by transwell migration assay. Scale bar: 100 μm. (**E**) Transwell invasion assay showed that overexpression of CAMSAP2 promoted the invasion in SW-620 and SW-480 cells. Scale bar: 100 μm. (F) Enforced overexpression of CAMSAP2 promoted the lung metastasis in colorectal cancer. Scale bar: 500 μm. **P < 0.01; ***P < 0.001.

To determine whether CAMSAP2 was involved in colorectal cancer cell migration and invasion, a CAMSAP2-expressing plasmid was transduced into SW-620, SW-480 and HCT-8 cells, and the expression of CAMSAP2 was determined by using qRT-PCR analysis and Western blot analysis, respectively. The results showed that the mRNA (Fig. 2A and Supplementary Fig. 1A) and protein (Fig. 2B and Supplementary Fig. 1B) levels of CAMSAP2 in two tested colorectal cancer cells were much higher in colorectal cancer cells transfected with CAMSAP2-expressing plasmids compared with those cells transfected with empty vector. Next, we performed wound healing assay to detect whether CAMSAP2 promotes colorectal cancer cell migration. The results showed that overexpression of CAMSAP2 upregulated the wound healing rate in all tested colorectal cancer cells, compared to the control group (Fig. 2C and Supplementary Fig. 1C). Moreover, transwell migration assay also revealed that ectopic expression of CAMSAP2 promoted the number of tumor cells migrated through the membrane (Fig. 2D and Supplementary Fig. 1D). To test whether CAMSAP2 influences the invasive ability of colorectal cancer cells, a transwell invasion assay was performed. As shown in Fig. 2E and Supplementary Fig. 1E, the number of tumor cells invaded through the matrigel-coated transwell inserts was increased by CAMSAP2 in all tested colorectal cancer cells. We next investigate whether CAMSAP2 promotes the metastasis of ESCC cells in nude mice. SW-620 cells with or without CAMSAP2 overexpression were intravenously injected into the nude mice via the lateral tail vein. Six weeks later, the lungs were dissected and fixed in Bouin’s solution. As shown in Fig. 2F, the number and size of metastatic nodules were upregulated in the lungs of CAMSAP2-overexpressing mice. Taken together, these findings demonstrated that CAMSAP2 promoted colorectal cancer metastasis in vivo.

### Knockdown of CAMSAP2 inhibited colorectal cancer metastasis

**Figure 3:**
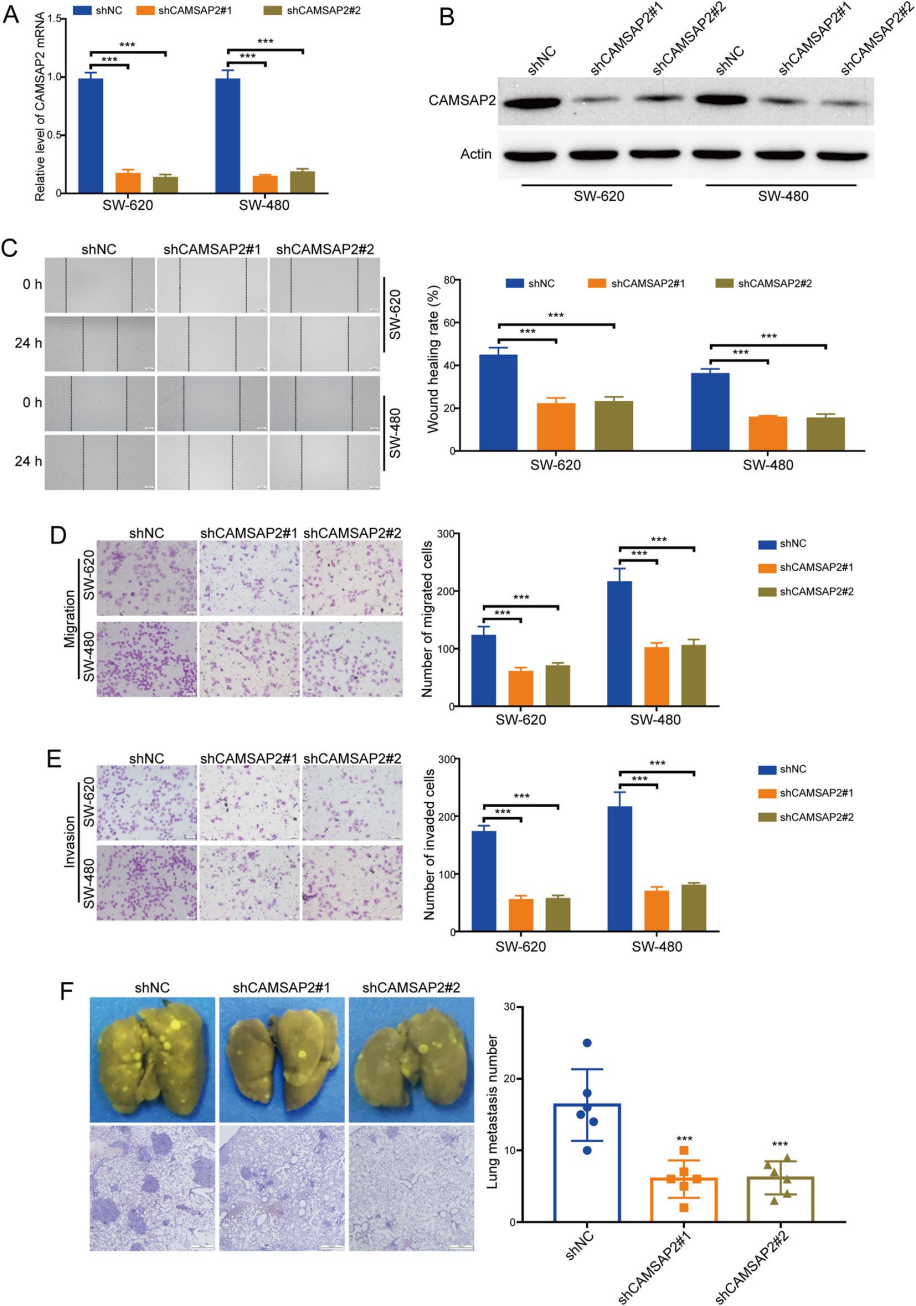
Knockdown of CAMSAP2 inhibited colorectal cancer cell migration and invasion. (**A**) The mRNA level of CAMSAP2 in SW-620 and SW-480 cells transduced with CAMSAP2 shRNAs (shCAMSAP2 #1 and shCAMSAP2 #2) or shNC was detected using qRT-PCR analysis. (**B**) Western blot analysis of the protein levels of CAMSAP2 in SW-620 and SW-480 cells transduced with CAMSAP2 shRNAs or shNC. (**C**) Silencing CAMSAP2 suppressed colorectal cancer cell migration measured using wound healing assay. Left: representative images of wound scratch. Right: histograms represent the analysis of the wound healing rate. Scale bar: 100 μm. (**D**) Knocking down CAMSAP2 hampered the migration of colorectal cancer cells detected by transwell migration assay. Scale bar: 100 μm. (**E**) Silencing CAMSAP2 suppressed the invasion of colorectal cancer cells examined using transwell invasion assay. Scale bar: 100 μm. (**F**) Silencing CAMSAP2 inhibited the lung metastasis of colorectal cancer in nude mice. Scale bar: 500 μm. ***P < 0.001.

Next, the expression of CAMSAP2 in SW-620 and SW-480 cells was stably silenced by using two shRNA targeting CAMSAP2 (shCAMSAP2#1 and shCAMSAP2#2). The results of qRT-PCR assay showed that the mRNA level of CAMSAP2 in both tested tumor cells was five times lower in response to CAMSAP2 lentivirual shRNAs than those cells transduced with shNC (Fig. 3A). Western blot analysis also revealed that the protein levels of CAMSAP2 in SW-620 and SW-480 cells were reduced in response to CAMSAP2 shRNAs (Fig. 3B). Next, we examined whether knockdown of CAMSAP2 inhibits colorectal cancer cell migration using wound healing assay. The results showed that knocking down CAMSAP2 decreased the migration speed of SW-620 and SW-480 cells (Fig. 3C). We also performed transwell migration assay to determine the role of CAMSAP2 in colorectal cancer cell migration. As illustrated in Fig. 3D, the migratory ability of SW-620 and SW-480 cells was suppressed by CAMSAP2 shRNAs. Subsequently, we tested whether silencing CAMSAP2 decreases the invasive ability of colorectal cancer cell using transwell invasion assay. The results showed that the invasive ability of SW-620 and SW-480 cells was hampered in response to CAMSAP2 downregulation (Fig. 3E). More importantly, the number and size of metastatic nodules were decreased in the lungs of CAMSAP2-depleted mice, compared to those of the shNC group (Fig. 3F). Taken together, these data confirmed that knockdown of CAMSAP2 hampered the metastatic ability of colorectal cancer.

### CAMSAP2 did not affect the growth of colorectal cancer cells

Subsequently, we determined whether CAMSAP2 promotes colorectal cancer cell growth by using MTT assay. Briefly, cells (1000 per well) were seeded in 96-well plates and incubated for 1 day, 2 days, 3 days, 4 days and 5 days. MTT solution (5 mg/ml) was added to each well and the plates were maintained at 37 °C for another 4 h. The Absorbance values were measured. The results showed that viabilities of cells transfected with CAMSAP2 plasmids were not changed in both SW-620 and SW-480 cells, compared with the control group (Supplementary Fig. 2A). Moreover, a similar phenomenon was observed in CAMSAP2-depleted colorectal cancer cells (Supplementary Fig. 2B). Furthermore, we also performed soft agar colony formation assay to determine the effect of CAMSAP2 on the growth of colorectal cancer cells. The results showed that overexpression (Supplementary Fig. 2C) or knockdown (Supplementary Fig. 2D) of CAMSAP2 hardly affected the number and size of colony formed in soft agar in both tested colorectal cancer cells. Collectively, these findings indicated that CAMSAP2 did not affect colorectal cancer cell growth.

### MMP-1 was crucial for CAMSAP2-mediated migration and invasion in colorectal cancer cells

**Figure 4:**
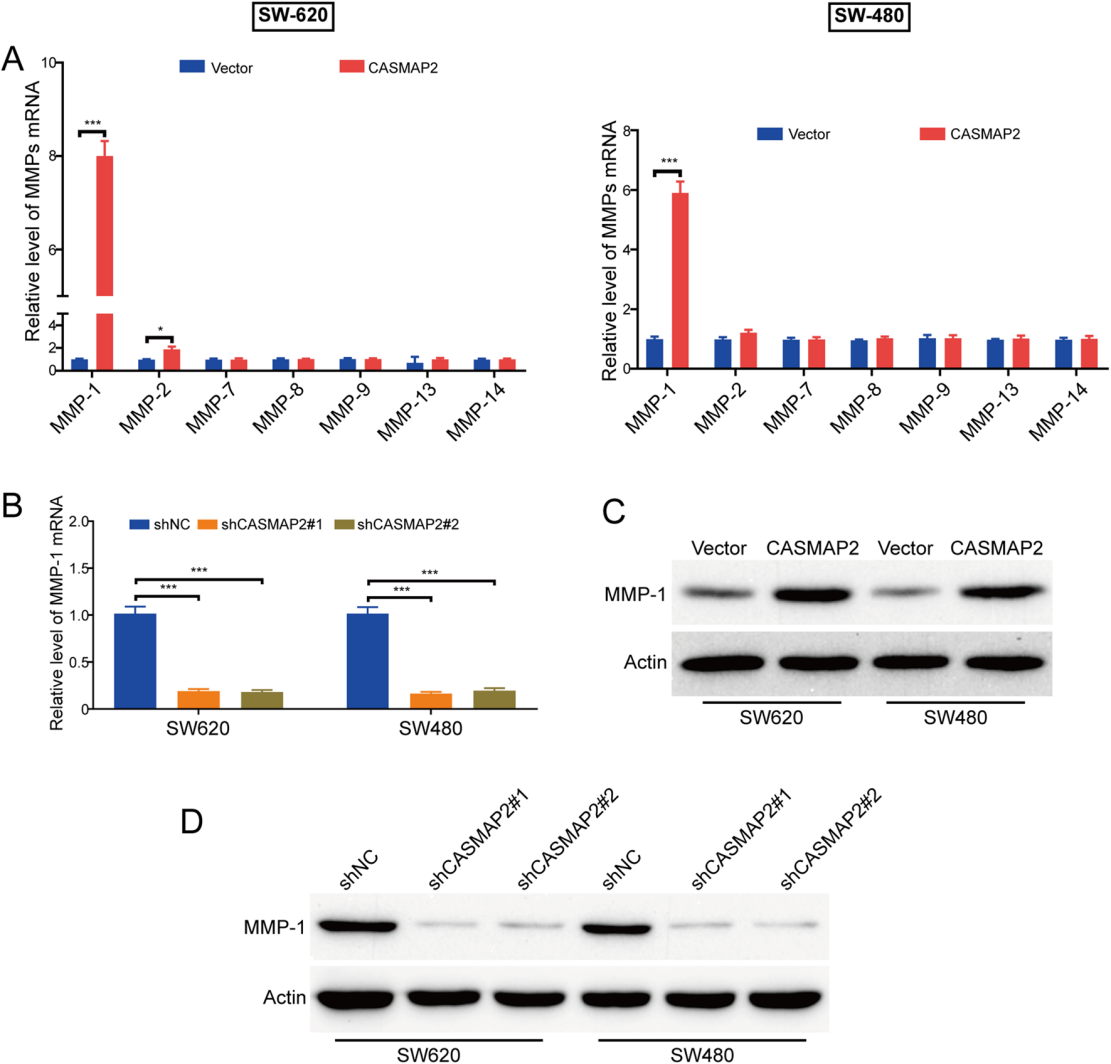
CAMSAP2 promotes MMP-1 expression in colorectal cancer cells. (**A**) qRT-PCR analyzed the mRNA level of MMP-1, MMP-2, MMP-7, MMP-8, MMP-9, MMP-13 and MMP-14 in SW-620 and SW-480 cells transfected with CAMSAP2-expressing plasmid or empty vector. (**B**) CAMSAP2 shRNA reduced the mRNA level of MMP-1 in SW-620 and SW-480 cells transduced with shRNAs against CAMSAP2 (shCAMSAP2#1 and shCAMSAP2#2). (**C**) Ectopic expression of CAMSAP2 promoted the protein level of MMP-1. (**D**) Western blot analysis showed that silencing CAMSAP2 inhibited MMP-1 expression in colorectal cancer cells. *P < 0.05; ***P < 0.001.

Matrix metalloproteinases (MMPs) is a family of zinc-containing endopeptidases, which play a crucial role in regulating tumor angiogenesis, invasion, and metastasis via remodeling and degradation of the extracellular matrix^20^. Several subtypes of MMPs, including MMP-1, MMP-2, MMP-7, MMP-8, MMP-9, MMP-13 and MMP-14, have been identified as potential biomarkers for colorectal cancer^3, 21–26^. Therefore, we analyzed whether CAMSAP2 affects the expression of these MMPs using qRT-PCR analysis. As shown in Fig. 4A, ectopic expression of CAMSAP2 hardly changed the mRNA levels of MMP-7, MMP-8, MMP-9, MMP-13 and MMP-14. Intriguingly, the mRNA level of MMP-1 was upregulated by CAMSAP2 in both SW-620 and SW-480 cells. Vice versa, knocking down CAMSAP2 by lentivirual shRNAs reduced the mRNA levels of MMP-1 in both tested colorectal cancer cells (Fig. 4B). Subsequently, we performed Western blot analysis to determine whether CAMSAP2 affects the protein levels of MMP-1. As illustrated in Fig. 4C, enforced overexpression of CAMSAP2 enhanced the expression of MMP-1 protein. On the contrary, the protein levels of MMP-1 were diminished by CAMSAP2 shRNAs, compared with the control group (Fig. 4D). Taken together, these data demonstrated that CAMSAP2 transcriptionally upregulated MMP-1 in colorectal cancer cells.

**Figure 5:**
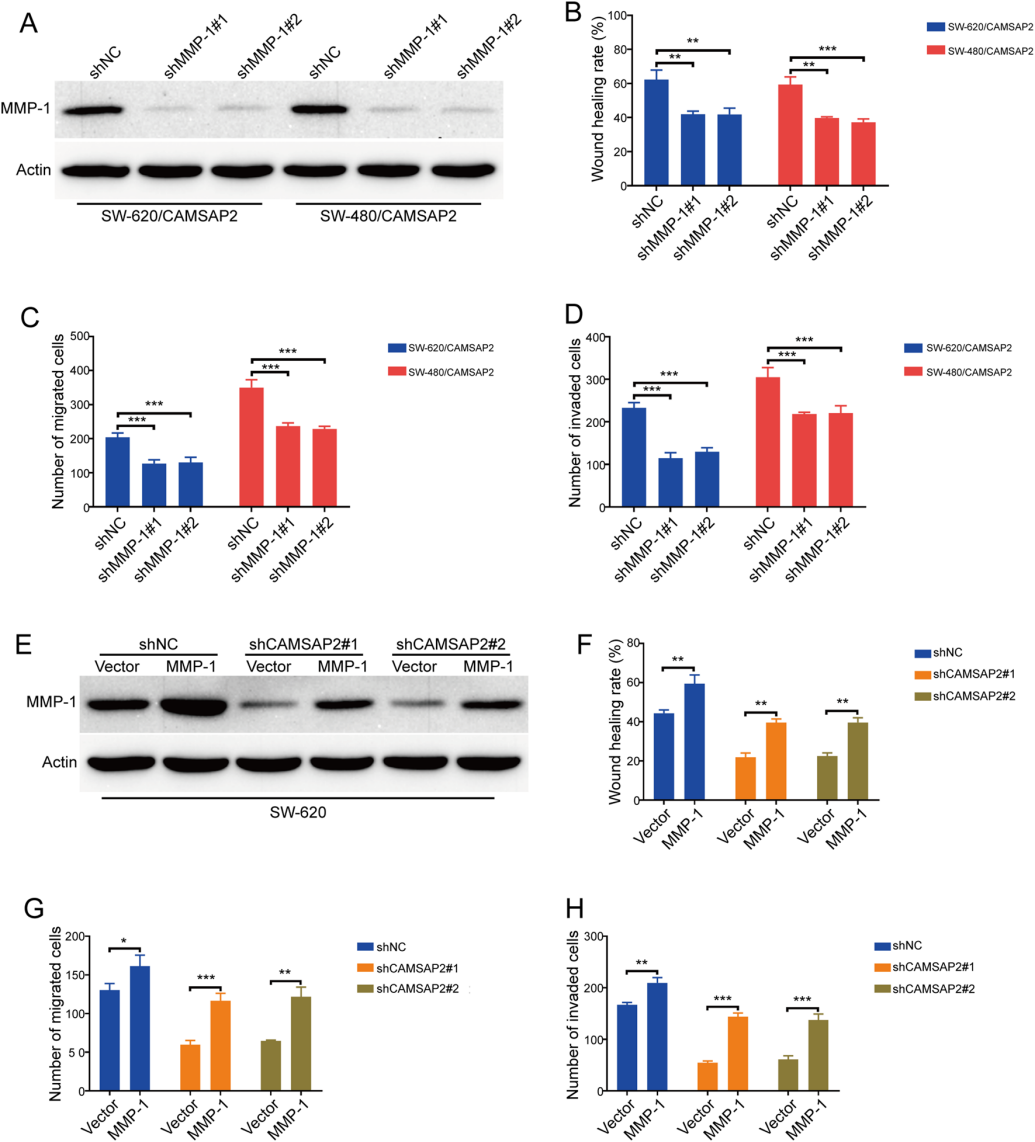
MMP-1 was essential for CAMSAP2-induced migration and invasion in colorectal cancer cells. (**A**) Western blot analysis showed that MMP-1 was silenced by shRNA against MMP-1 in CAMSAP2-overexpessing SW-620 cells. (**B**) Wound healing scratch assay showed ectopic expression of MMP-1 attenuated CAMSAP2 shRNA-reduced migration in SW-620 and SW-480 cells. Left: representative images of wound scratch. Right: histograms represent the analysis of the wound healing rate. (**C**) Transwell migration assay showed overexpression of MMP-1 attenuated the inhibition of CAMSAP2 shRNA on colorectal cancer cell migration. (**D**) Enforced overexpression of MMP-1 restored the invasion-attenuated by CAMSAP2 shRNAs in colorectal cancer cells. (**E**) Western blot analysis of MMP-1 expression in CAMSAP2-silenced SW-620 and SW-480 cells transduced with MMP-1 plasmid (MMP-1) or empty vector (Vector). (**F**) Wound healing assay showed silencing MMP-1 attenuated CAMSAP2-induced migration in colorectal cancer cells. Left: representative images of wound scratch. Right: histograms represent the analysis of the wound healing rate. (**G**) Transwell migration assay showed knockdown of MMP-1 attenuated the CAMSAP2-induced migration in SW-620 cells. (H) Knockdown of MMP-1 inhibited the colorectal cancer cell invasion induced by CAMSAP2 overexpression. *P < 0.05; **P < 0.01; ***P < 0.001.

To further investigate whether MMP-1 is involved in CAMSAP2-mediated colorectal cancer cell migration and invasion, two lentiviral shRNAs against MMP-1 was transduced into colorectal cancer cells, the knockdown efficiency was measured using Western blot analysis. The results showed that the protein levels of MMP-1 in SW-620/CAMSAP2 and SW-480/CAMSAP2 cells were reduced in response to MMP-1 shRNAs treatment, compared with those cells transduced with shNC (Fig. 5A). As expect, wound healing assay revealed that knocking down MMP-1 decreased the migration speed of SW-620/CAMSAP2 and SW-480/CAMSAP2 cells (Fig. 5B). In addition, transwell migration and invasion assays also showed that silencing MMP-1 inhibited the migration and invasion induced by CAMSAP2 in both tested colorectal cancer cells (Fig. 5C,D). Next, we performed wound healing assay to investigate whether overexperesson of MMP-1 reduces the inhibitory effects of CAMSAP2 shRNAs on colorectal cancer cell migration. Intriguingly, overexpression of MMP-1 rescued the migration speed inhibited by CAMSAP2 shRNAs in both SW-620 and SW-480 cells (Fig. 5E,F). Furthermore, the CAMSAP2 shRNAs-inhibited migration and invasion was reversed in response to MMP-1 overexpression, as observed in transwell migration and invasion assays (Fig. 5G,H). These findings suggested that MMP-1 played a critical role in CAMSAP2-mediated colorectal cancer cell migration and invasion.

### CAMSAP2 promoted the expression of MMP-1 through activation of JNK/c-Jun signaling pathway

**Figure 6:**
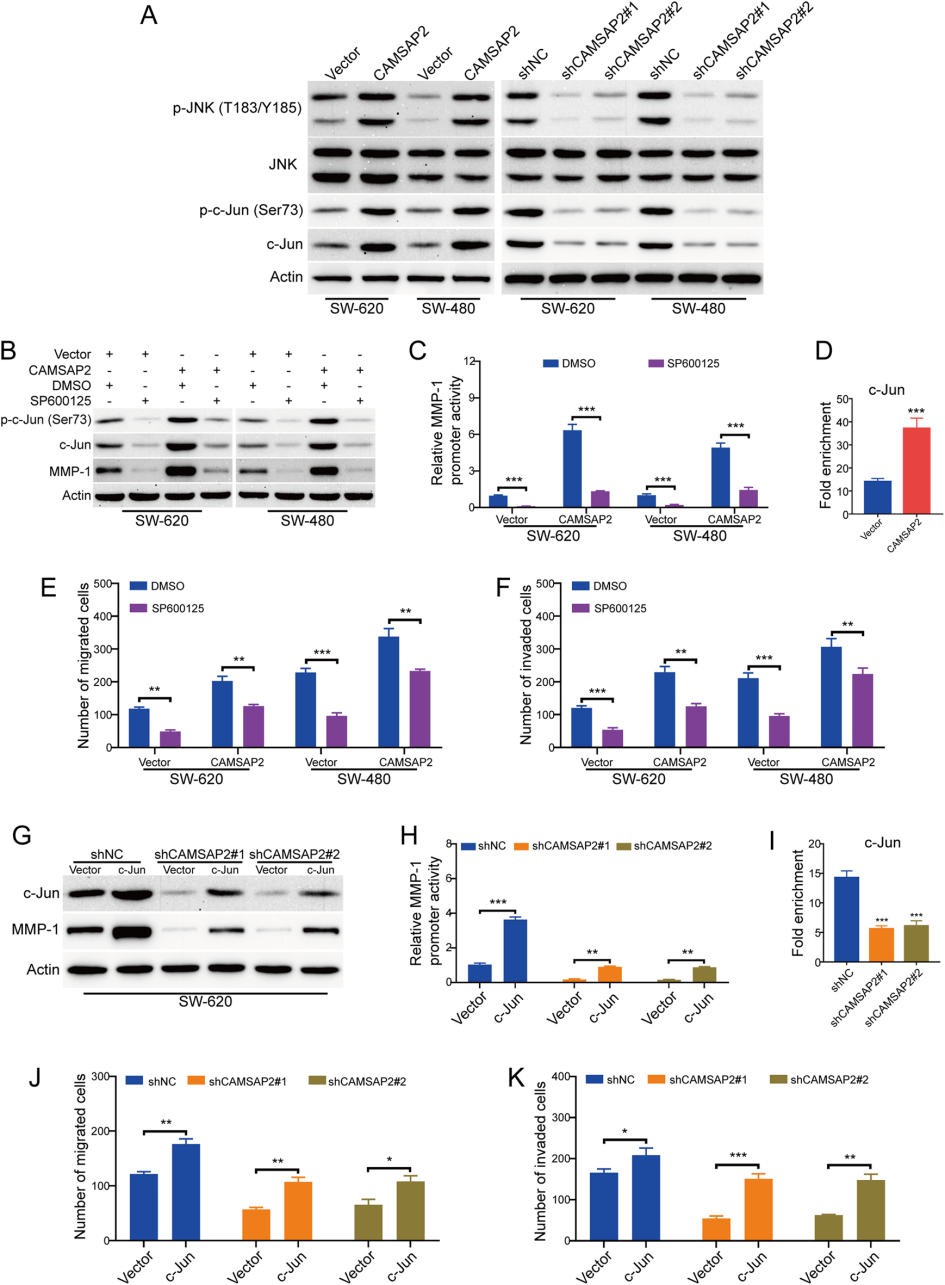
CAMSAP2 enhanced the expression of MMP-1 through activation of JNK/c-Jun signaling pathway. (**A**) Western blot analysis of p-JNK (T183/Y185), JNK, p–c-Jun (Ser73) and c-Jun in CAMSAP2 expressing or depleted colorectal cancer cells. (**B**) Targeting JNK by SP600125 reduced the expression of MMP-1 induced by CAMSAP2 in colorectal cancer cells. (**C**) Representative luciferase reporter assay from colorectal cancer cells transfected with an MMP-1 luciferase reporter, in the presence or absence of SP600125 (10 μM). Promoter activity was measured using a dual-luciferase system. Data are presented as relative to the DMSO control. (**D**) ChIP analysis of c-Jun occupancy on the promoter of MMP-1 in SW-620 cells. Fold enrichments normalized to IgG are presented. The values are means and SDs of three independent experiments. (**E,F**) Transwell migration (**E**) and invasion (**F**) assay revealed that SP600125 attenuated the migration and invasion induced by CAMSAP2 overexpression in colorectal cancer cells. (**G**) Western blot analysis of c-Jun and MMP-1 in CAMSAP2-depleted SW-620 cells transduced with c-Jun plasmid or empty vector. (**H**) Luciferase reporter assay revealed that ectopic expression of c-Jun restored the transcription activity of MMP-1-reduced by CAMSAP2 shRNAs in SW-620 cells. Promoter activity was measured using a dual-luciferase system. Data are presented as relative to the control group (shNC + Vector). (**I**) ChIP experiment showed that knockdown of CAMSAP2 led to loss of recruitment of c-Jun on the MMP-1 gene promoter of in SW-620 cells. (**J,K**) Transwell migration (**J**) and invasion (**K**) assay showed that enforced expression of c-Jun rescued the migration and invasion attenuated by CAMSAP2 shRNAs in SW-620 cells. *P < 0.05; **P < 0.01; ***P < 0.001.

Next, we asked how CAMSAP2 regulates MMP-1 in colorectal cancer cells. Previous studies have demonstrated that the expression of MMP-1 is transcriptionally upregulated by c-Jun, a well-known downstream substrate in JNK pathway^27, 28^. In addition, Li et al. recently reported that CAMSAP2 promoted hepatocellular carcinoma invasion and metastasis through activation of JNK/c-Jun signaling pathway^11^. Therefore, we hypothesized that CAMSAP2 may regulate MMP-1 transcription in colorectal cancer cells by stimulating JNK/c-Jun signaling pathway. To confirm this hypothesis, we firstly performed Western blot analysis to examine whether CAMSAP2 regulates the activation of JNK/c-Jun signaling pathway. As shown in Fig. 6A, overexpression of CAMSAP2 upregulated, whereas knocking down CAMSAP2 decreased the protein levels of p-JNK (T183/Y185), p–c-Jun (Ser73) and c-Jun. Next, we examined whether targeting JNK by SP600125 affects the expression of MMP-1 in colorectal cancer cells. The results from Western blot analysis showed that SP600125 treatment suppressed the expression of p–c-Jun (Ser73), c-Jun and MMP-1 in both tested colorectal cancer cells (Fig. 6B); interestingly, the increase expression of p–c-Jun (Ser73), c-Jun and MMP-1 induced by CAMSAP2 was also attenuated in response to SP600125 treatment (Fig. 6B). Additionally, upon treatment with SP600125, the promoter activity of MMP-1 induced by CAMSAP2 upregualtion was inhibited in both tested cell lines of colorectal cancer (Fig. 6C). ChIP analysis also showed that overexpression of CAMSAP2 enhanced the occupancy of c-Jun on the MMP-1 gene promoter region in SW-620 cells (Fig. 6D). Subsequently, we performed transwell assay to test whether targeting JNK hampered CAMSAP2-mediated migration and invasion in colorectal cancer cells. The results showed that the migration (Fig. 6E) and invasion (Fig. 6F) induced by CAMSAP2 overexpression was inhibited by SP600125 in both tested colorectal cancer cells. To investigate if c-Jun was required for MMP-1 expression-induced by CAMSAP2 in these cells; a c-Jun expressing plasmid was tranduced into CAMSAP2-depleted SW-620 cells. As illustrated in Fig. 6G, Enforced overexpression of c-Jun attenuated the inhibitory effects of CAMSAP2 shRNAs on MMP-1 expresison or promoter activity (Fig. 6G,H). In addition, knocking down CAMSAP2 led to loss of recruitment of c-Jun on the MMP-1 gene promoter of in SW-620 cells (Fig. 6I). More importantly, ectopic expression of c-Jun in SW-620 cells rescued the migration (Fig. 6J) and invasion (Fig. 6K) reduced by CAMSAP2 shRNAs. Taken together, our results confirmed that CAMSAP2 enhanced the expression of MMP-1 through activation of JNK/c-Jun signaling pathway, and thereby promoted the migration and invasion of colorectal cancer cells.

## Discussion

CAMSAP2 belongs to an important family member of CAMSAPs and has been demonstrated as an oncogene in HCC^4^. However, little is known about its expression and potential function in colorectal cancer. In this study, we discovered that the expression of CAMSAP2 was highly elevated in colorectal cancer tissues and cell lines. Ectopic expression of CAMSAP2 significantly enhanced the migration, invasion and metastasis of colorectal cancer cells. By gain- and loss-of function studies, we demonstrated that MMP-1 was essential for the migration and invasion induced by CAMSAP2. Mechanistically, CAMSAP2 activated the JNK/c-Jun signaling pathway, and thereby upregulated the transcription of MMP-1. Our findings indicate that CAMSAP2 is a promising therapeutic target for patients with metastatic colorectal cancer. To the best of our knowledge, this is the first study to examine the expression and function of CAMSAP2 in colorectal cancer.

Matrix metalloproteinases (MMPs) is a large family of at least 25 zinc-dependent endopeptidases, which are capable of degrading and shedding all components of the extracellular matrix (ECM) including growth factors, receptors and cell adhesion molecules^29^. According to their structural features, MMPs can be categorized as collagenases, gelatinases, stromelysins, membrane-type and matrilysins^29^. Increasing studies have confirmed the important function of MMPs to promote neoangiogenesis, invasion and metastasis in various malignant tumors including colorectal cancer^22, 30^. MMP-1, as an important member of the collagenases, plays a critical role in degrading ECM, especially type I, II and III collagens, the major components of the interstitial stroma^29^. In colorectal cancer, high MMP-1 expression not only significantly correlates with hematogenous metastasis, but also correlates with the depth grading of tumor invasion, tumor growth pattern, the presence of lymphatic invasion, venous invasion, neural invasion, lymph node metastasis, hepatic metastasis, and increasing stages of Dukes' classification^31, 32^. On the contrary, inhibition of MMP-1 by chemical inhibitors or neutralizing antibodies drastically reduced the abilities of migration and invasion in colorectal cancer cells^33^. Consistently with these findings, in the present study, we found that MMP-1 played an important role in CAMSAP2-induced migration and invasion in colorectal cancer cells, which was based on the following facts: (1) Overexpression of CAMSAP2 promoted, whereas silencing CAMSAP2 hampered the migration and invasive ability of colorectal cancer cells; (2) Ectopic expression of CAMSAP2 increased, while knocking down CAMSAP2 reduced MMP-1 expression at both the mRNA and protein levels; (3) Silencing MMP-1 attenuated CAMSAP2-induced migration and invasion; whereas enforced overexpression of MMP-1 almost completely depleted the inhibitory effects of CAMSAP2 shRNAs on the capabilities of migration and invasion in colorectal cancer cells.

Aberrant activation of JNK/c-Jun signaling pathway has been discovered in various malignancies including colorectal cancer^14, 34^. As a critical downstream transcription factor in JNK signaling, c-Jun is essential for colorectal cancer cell migration, invasion and metastasis^13, 15^. In this study, we demonstrated that ectopic expression of CAMSAP2 drastically upregulated the expression of p-JNK (T183/Y185), p–c-Jun (Ser73) and c-Jun. Moreover, inhibition of JNK/c-Jun signaling by a specific chemical inhibitor (SP600125) downregulated the transcription activity of MMP-1 and inhibited the capabilities of migration and invasion mediated by CAMSAP2 in colorectal cancer cells, indicating JNK/c-Jun signaling is essential for CAMSAP2-induced metastasis in colorectal cancer. Consistent with our findings, Li et al. demonstrated that CAMSAP2 directly activates JNK/c-Jun signaling to drive hepatocellular carcinoma metastasis^9^. Taken together, these findings from us and others suggested that JNK/c-Jun signaling pathway plays an important role in CAMSAP2-induced metastasis in various malignant tumors.

## Conclusion

In summary, our study revealed that CAMSAP2 promoted the migration, invasion and metastasis of colorectal cancer cells through activation of JNK/c-Jun/MMP-1 signaling pathway, suggesting CAMSAP2 is a potential therapeutic target for metastatic colorectal cancer.

## Supplementary Information

Supplementary Information 1.

Supplementary Information 2.

## Data Availability

All data generated or analyzed during this study are included in this published article.
