# Supplementary figures and images for: CAMSAP2 promotes colorectal cancer cell migration and invasion through activation of JNK/c-Jun/MMP-1 signaling pathway
Source: Sci Rep. 2022 Oct 7;12:16899. doi: 10.1038/s41598-022-21345-7 (PMC9546856; doi:10.1038/s41598-022-21345-7)

Figure 1F

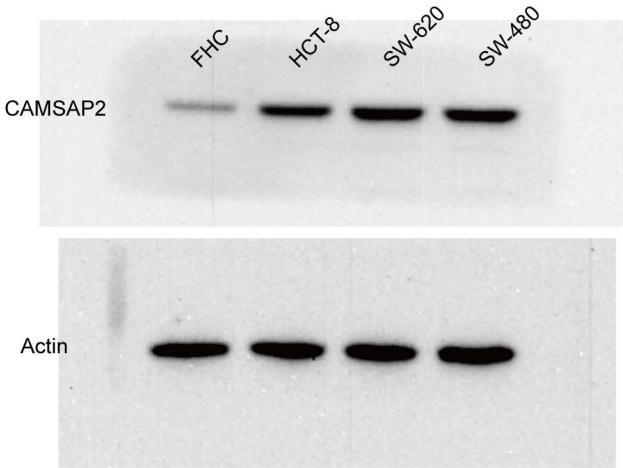

Figure 2B

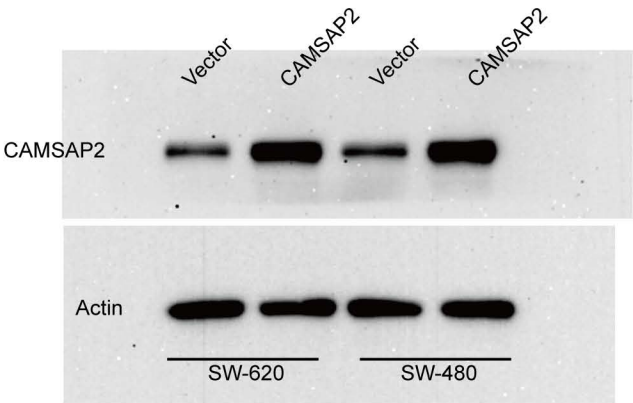

Figure 3B

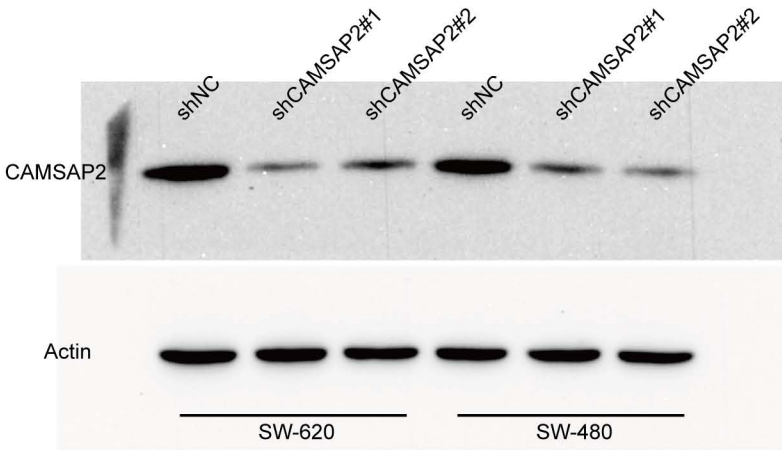

Figure 4

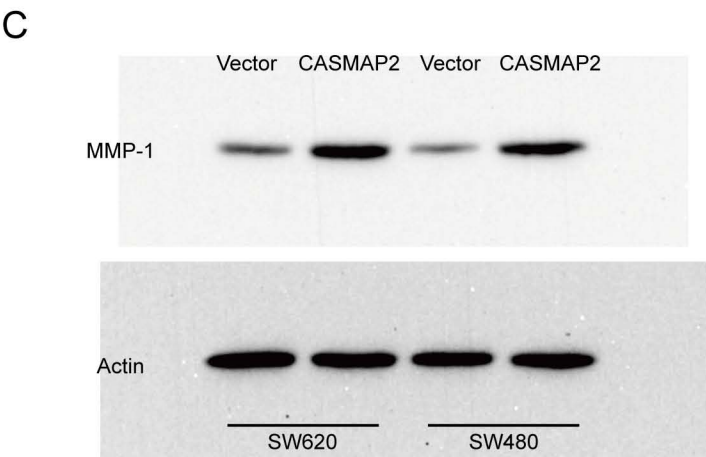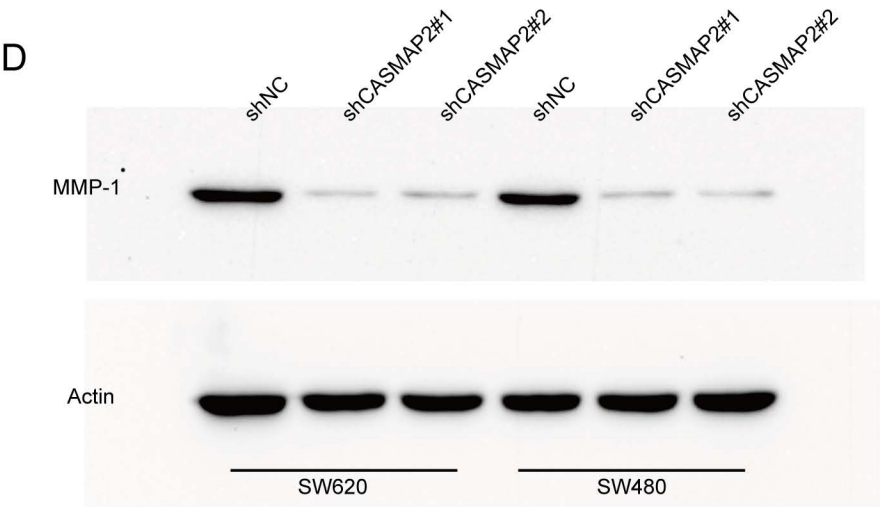

Figure 5

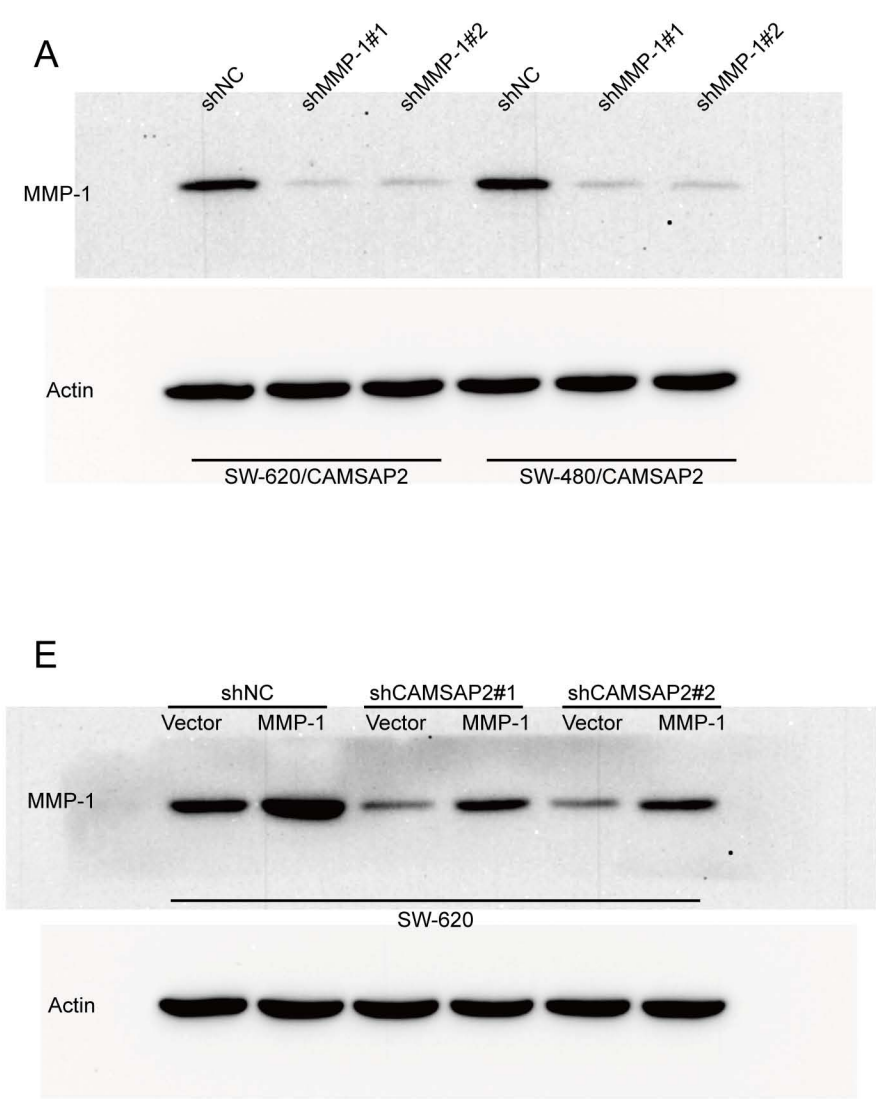

Figure 6

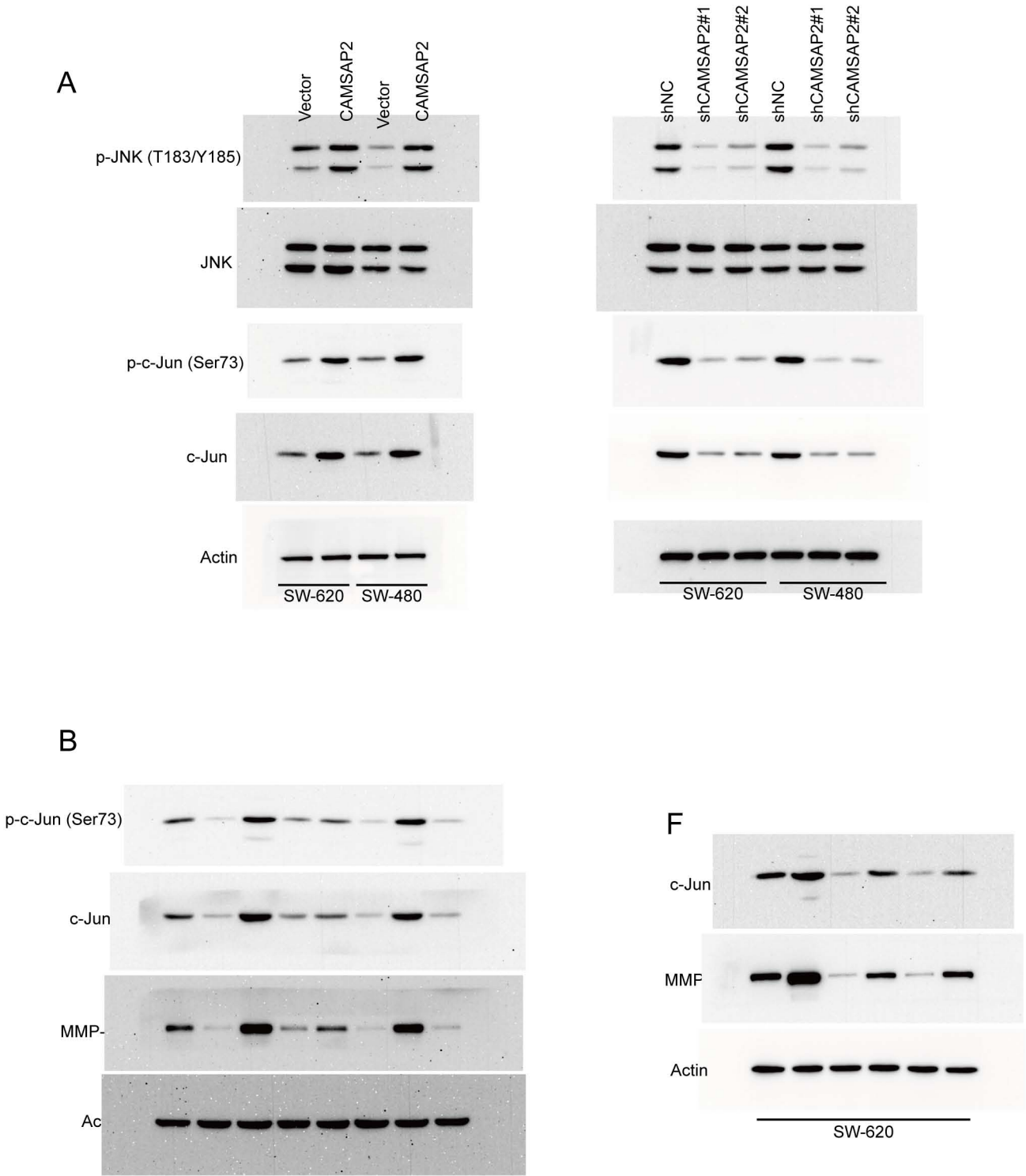

supplementary Figure S1

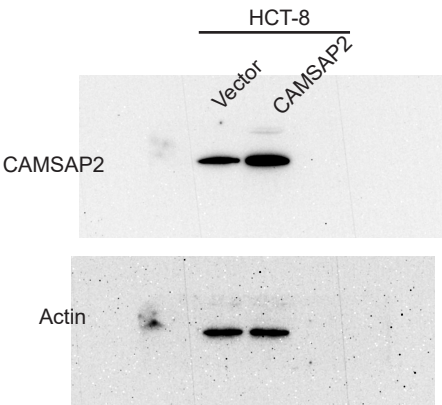

Supplement: Supplementary file 1 — Supplementary Information 1. [file 41598_2022_21345_MOESM1_ESM.pdf]
