# Supplementary material for: CAMSAP2 promotes colorectal cancer cell migration and invasion through activation of JNK/c-Jun/MMP-1 signaling pathway
Source: Sci Rep. 2022 Oct 7;12:16899. doi: 10.1038/s41598-022-21345-7 (PMC9546856; doi:10.1038/s41598-022-21345-7)
Supplement: Supplementary file 2 — Supplementary Information 2. [file 41598_2022_21345_MOESM2_ESM.pdf]

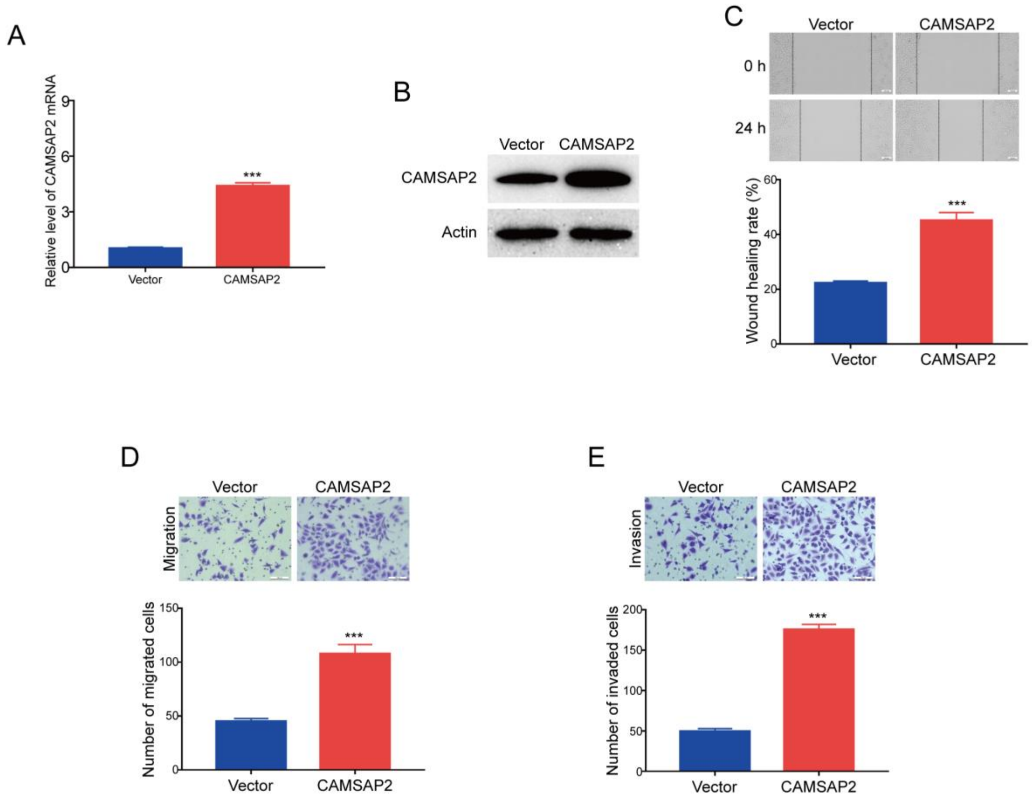

Supplementary Figure S1 Overexpression of CAMSAP2 promoted migration and invasion in HCT-8 cells. (A) qRT-PCR analysis of CAMSAP2 mRNA in HCT-8 cells transduced with CAMSAP2 or control plasmid (Vector). (B) Immunoblotting analysis validated that CAMSAP2 was overexpressed in HCT-8 cells transduced with CAMSAP2 plasmid. (C) Wound healing assay showed overexpression of CAMSAP2 enhanced cell migration in HCT-8 cells. Scale bar: 100 μm. (D) Overexpression of CAMSAP2 promoted the migration of HCT-8 cells examined by transwell migration assay. Scale bar: 100 μm. (E) Transwell invasion assay showed that overexpression of CAMSAP2 promoted the invasion in HCT-8 cells. Scale bar: 100 μm. ****P* < 0.001.

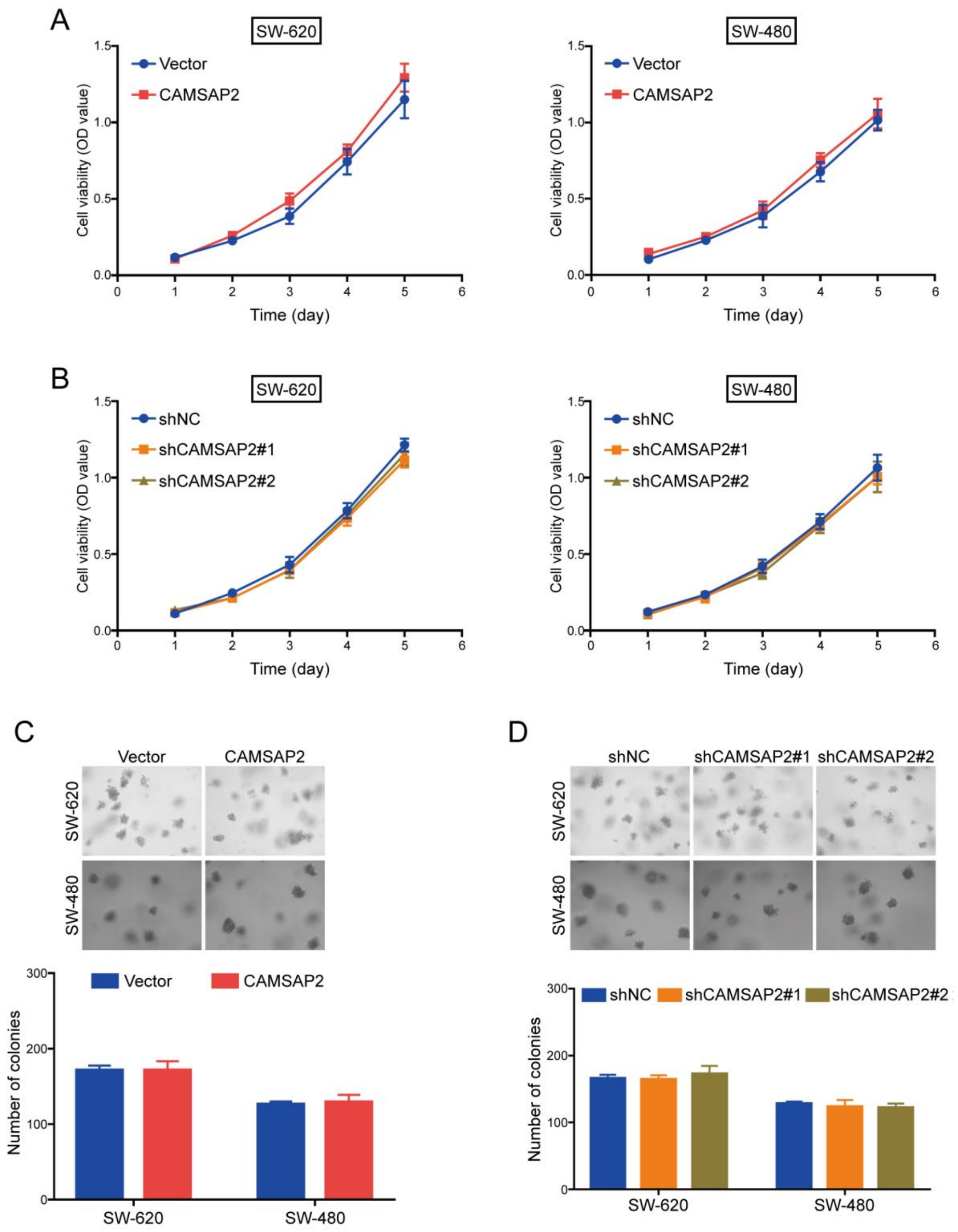

Supplementary Figure S2 Overexpression or knockdown of CAMSAP2 did not affect the growth of colorectal cancer cells. (A) MTT assay was used to measure the viabilities of cells with CAMSAP2 overexpression. (B) The viabilities of colorectal cancer cells with stable CAMSAP2 depletion were measured by MTT assay. (C) Representative images (*left*) and statistics (*right*) of colony formation in soft agar assay (n=6) in SW-620 and SW-480 cells stably transfected with CAMSAP2 or empty vector. (D) Silencing CAMSAP2 did not reduce the number of colony formation in soft agar.

Supplementary Table S1 The clinic-pathological factors and expression of CAMSAP2 in 76 colorectal cancer cases

Characteristics	Number of cases	CAMSAP2 expression		*P* value
		Low (38)	High (38)	
Age (year)				
< 60	32	20	12	0.1032
≥ 60	44	18	26	
Gender				
Female	36	21	15	0.2506
Male	40	17	23	
Tumor invasion depth				
T1-2	48	30	18	**0.0099**
T3-4	28	9	21	
Lymph node metastasis				
N0	45	29	16	**0.0359**
N1 + N2	31	12	19	
Distant metastasis				
M0	50	33	17	**0.0042**
M1 + M2	26	8	18	
TNM stage				
I + II	52	33	19	0.0249
III + IV	24	8	16	

[a]Based on Pearson $\chi 2$ test (Fisher exact test was used when needed).

Supplementary Table S2 Primer sequences used for qRT-PCR in this study

Genes	Sense primer	Antisense primer
CAMSAP2	5'-GGAAACTGGTTCCAGCTCGTT-3'	5'-GCCATCTGTCCCATCCTTCA-3'
MMP-1	5'-ATGCACAGCTTTCCTCCACT-3'	5'-TTCCCAGTCACTTTCAGCCC-3'
MMP-2	5'-GATACCCCTTTGACGGTAAGGA-3'	5'-CCTTCTCCCAAGGTCCATAGC-3'
MMP-7	5'-GCTACAGTGGGAACAGGCTC-3'	5'-TGGCCCATCAAATGGGTAGG-3'
MMP-8	5'-TTGGGTTGAATGTGACGGGG-3'	5'-GGGTATAGTTTCGAATCCTGTAGGT-3'
MMP-9	5'-GGGACGCAGACATCGTCATC-3'	5'-TCGTCATCGTCGAAATGGGC-3'
MMP-13	5'-ACTGAGAGGCTCCGAGAAATG-3'	5'-GAACCCCGCATCTTGGCTT-3'
MMP-14	5'-GGACCTACGTACCCACACAC-3'	5'-GCCTCATGGCCTTCATGGT-3'
GAPDH	5'-CCATCACCATCTTCCAGGAG-3'	5'-CCTGCTTCACCACCTTCTTG-3'

Supplementary Table S3 Primer sequences used for ChIP in this study

Genes	Sense primer	Antisense primer
MMP-1	5'- GCAACACCAAGTGATTCCAA-3'	5'- CCTTTGTCTTCTTTCTCAGTGCAAG-3'
